# *Heterobasidion annosum* Induces Apoptosis in DLD-1 Cells and Decreases Colon Cancer Growth in In Vivo Model

**DOI:** 10.3390/ijms21103447

**Published:** 2020-05-13

**Authors:** Anna Sadowska, Ewa Zapora, Diana Sawicka, Katarzyna Niemirowicz-Laskowska, Arkadiusz Surażyński, Katarzyna Sułkowska-Ziaja, Katarzyna Kała, Marcin Stocki, Marek Wołkowycki, Sławomir Bakier, Anna Pawlik, Magdalena Jaszek, Bożena Muszyńska, Halina Car

**Affiliations:** 1 Department of Experimental Pharmacology, Medical University of Białystok, Szpitalna 37, 15-295 Bialystok, Poland; diana.sawicka@umb.edu.pl (D.S.); katia146@wp.pl (K.N.-L.); halina.car@umb.edu.pl (H.C.); 2 Institute of Forest Sciences, Bialystok University of Technology, Wiejska 45E, 15-351 Bialystok, Poland; e.zapora@pb.edu.pl (E.Z.); m.stocki@pb.edu.pl (M.S.); m.wolkowycki@pb.edu.pl (M.W.); s.bakier@pb.edu.pl (S.B.); 3 Department of Medicinal Chemistry, Medical University of Bialystok, Mickiewicza 2D, 15-222 Bialystok, Poland; arkadiusz.surazynski@umb.edu.pl; 4 Department of Pharmaceutical Botany, Jagiellonian University Medical College, Medyczna 9, 30-688 Krakow, Poland; katarzyna.sulkowska-ziaja@uj.edu.pl (K.S.-Z.); kat3kala@gmail.com (K.K.); muchon@poczta.fm (B.M.); 5 Department of Biochemistry and Biotechnology, Maria Curie Sklodowska University, Akademicka 19, 20-033 Lublin, Poland; anna.pawlik@poczta.umcs.lublin.pl (A.P.); magdalena.jaszek@poczta.umcs.lublin.pl (M.J.)

**Keywords:** *Heterobasidion annosum*, colon cancer, apoptosis, active substances, mouse model, toxicity

## Abstract

Application of substances from medicinal mushrooms is one of the interesting approaches to improve cancer therapy. In this study, we commenced a new attempt in the field of *Heterobasidion annosum* (Fr.) Bref. sensu lato to further extend our knowledge on this basidiomycete fungus. For this purpose, analysis of the active substances of *Heterobasidion annosum* methanolic extract and also its influence on colorectal cancer in terms of in vitro and in vivo experiments were performed. In vivo studies on mice were conducted to verify its acute toxicity and to further affirm its anticancer potential. Results indicated that all the most common substances of best known medicinal mushrooms that are also responsible for their biological activity are present in tested extracts. In vitro tests showed a high hemocompatibility and a significant decrease in viability and proliferation of DLD-1 cells in a concentration-dependent manner of *Heterobasidion annosum* extract. The studies performed on xenograft model of mice showed lower tendency of tumor growth in the group of mice receiving *Heterobasidion annosum* extract as well as mild or moderate toxicity. Obtained results suggest beneficial potential of *Heterobasidion annosum* against colon cancer as cytotoxic agent or as adjuvant anticancer therapy.

## 1. Introduction

*Heterobasidion annosum* (Fr.) Bref. *sensu lato* (s.l.) (Basidiomycota) is considered to be one of the most serious pathogens that cause both root and butt rot in conifer forests in the northern boreal and temperate regions of the world [1,2,3,4,5]. *H. annosum* s.l. species complex comprises five intersterile groups (IGs): *Heterobasidion annosum sensu stricto* (s.s.), *Heterobasidion parviporum, Heterobasidion abietinum, Heterobasidion irregulare, and Heterobasidion occidentale*, and they show different host preferences [5,6,7,8]. *Heterobasidion* sp. infect trees through wounds and newly cut stumps and grow within the trunk and roots of live and dead trees, causing white rot decay. The fungus is able to degrade wood due to secretion of many enzymes [9,10,11,12].

The most accurate data on chemical composition (total of 33 compounds) of five *Heterobasidion species* (*H. annosum s.s., H. irregulare, H. parviporum, H. abietinum, and H. occidentale*) was presented by Hansson. Importantly, the secondary metabolite profile of the five species of *H. annosum* s.l. is not identical [13].

Colorectal cancer (CRC) is one of the most common cancers with the highest morbidity in Australia, New Zealand, North America, and Europe. Differences in the incidence of the disease can be associated with different genetic susceptibility, environmental factors, and diet. Among the environmental factors predisposing to the development of colorectal cancer, attention is paid particularly to potentially modifiable factors such as obesity, diabetes, alcohol abuse, smoking, lack of physical activity, and a high-fat diet with a large content of red meat. For this reason, the incidence of colon cancer is constantly increasing.

Moreover, it is established that the development of the drug-resistance phenomena remains one of the deadlocks for the low survival rates of CRC patients. Challenge for future medicine study is to search for new active agents that can effectively inhibit the progression of cancer disease without disrupting the function of normal tissues [14,15].

More than 14,000 species of mushroom have been identified so far, although the actual number maybe even ten times greater. In addition, it has been evaluated that around 5% of mushrooms might be therapeutically useful [16]. Due to the presence of biologically active compounds, mushrooms have drawn attention in the field of medicine [17].

Numerous researches indicate a high therapeutic activity of extracts and compounds derived from polypore mushrooms. Medicinal mushrooms have been extensively used in Asian medicine as a remedy against various disorders, including cancer. Among all the known species of mushrooms, 650 possess documented medicinal properties and about 20 are currently in clinical use. Their antitumor, antibacterial, antifungal, antiviral, anti-inflammatory, anti-allergic, and immunomodulatory activities have been confirmed in the experimental studies [18,19,20]. The mushrooms with proven anticancer activity belong to the genus *Phellinus, Pleurotus, Agaricus, Ganoderma, Clitocybe, Antrodia, Trametes, Cordyceps, Xerocomus, Calvatia, Schizophyllum, Flammulina, Suillus, Inonotus, Inocybe, Funlia, Lactarius, Albatrellus, Russula, and Fomes* [21]. Extracts from these mushrooms contain bioactive compounds, including proteins, polysaccharides, glycosides, phenols, tocopherols, carotenoids, flavonoids, and organic acids able to inhibit mitosis and angiogenesis, induce apoptosis, and restrain proliferation of neoplastic cells. Disease treatment with substances extracted from mushrooms has been raised to the scientific rank and named mycotherapy [22].

Over the past decades, *H. annosum* s.l. has been the target of numerous scientific papers, which makes it one of the most extensively studied forest mushrooms [23]. Nevertheless, until now *H. annosum* s.s. has not been examined in terms of anticancer properties. There are no data concerning any medical use of *H. annosum* s.s. The presented results have indicated for the first time that the extract of *H. annosum* s.s. possesses cytotoxic activity against colorectal cancer cells.

In the present work, chemical composition profile of *Heterobasidion annosum* (HA) was analyzed. Moreover, the influence of HA methanolic extract on colorectal cancer cells in terms of in vitro and in vivo experiments was investigated.

## 2. Results and Discussion

### 2.1. Taxonomic Identification of M-0969 Strain Classifies to HA

To determine the phylogenetic position of the tested fungal strain, a 636 bp fragment of the ITS region was obtained from PCR with ITS1 and ITS4 primers and followed by direct sequencing. The sequence of this product revealed over 99% identity of M-0969 strain to HA, as shown in the NCBI-BLAST search system. The following GenBank accession number was assigned to the nucleotide sequence determined in this study: MK395162—HA strain M-0969 small subunit ribosomal RNA gene, partial sequence; internal transcribed spacer 1, 5.8S ribosomal RNA gene, and internal transcribed spacer 2, complete sequence; and large subunit ribosomal RNA gene, partial sequence.

### 2.2. Chemical Characteristic of M-0969 Strain Determine Their Biological Properties

**Figure 1:**
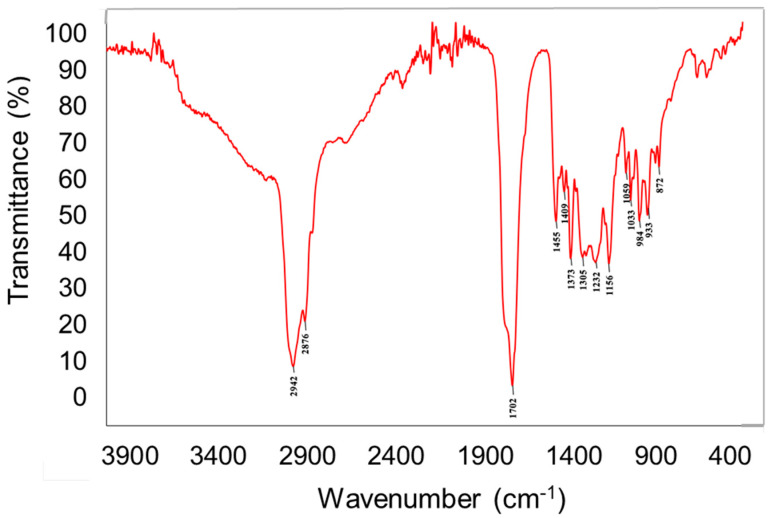
FTIR spectra of *Heterobasidion annosum* extract.

The chemical analysis of the crude extract was performed via engagement of different chemical techniques including spectral–FTIR (Fourier-transform infrared spectroscopy) and spectroscopic–AAS (atomic absorption spectroscopy) as well as chromatographic analysis including GC-MS and HPLC. It is very important due to the fact that there is evidence that the bioactive potential of the tested mushroom is determined by the chemical composition and has direct effects on their anticancer activity. The FTIR spectroscopy indicated the presence of intense bands at 2940–2840 cm^−1^, which corresponds to the stretching mode of C-H bonds. The medium size bands at 1460–1445 cm^−1^ and 1380–1370 cm^−1^ can be ascribed to the C–H deformations modes. Intense signals at 1740–1700 cm^−1^ were characterized as C=O vibrations. The signals at 1245–1010 cm^−1^ can be attributed to the C-O and C-N different types of vibrations (Figure 1). These signals are associated with the presence of different compounds, including carbohydrates, sterols, carboxylic acid, amino acids, etc., which were deeply characterized by other techniques and described in the next section. Interesting is the presence of the peak at 1246 cm^−1^, which is also characteristic for succinate and so-called “Baltic amber shoulder”. However, aforementioned signal might be also prescribed for N-Ar band in tryptophan molecules. This can indicate the presence of plant resin compounds that might have been absorbed by the fungus from its host.

Preliminary chemical analysis of the extract using GC-MS techniques showed the presence of compounds belonging to the groups carbohydrates (6.82% of TIC), sterols (1.82% of TIC), and carboxylic acids (1.74% of TIC) (Table 1 and Figure S1). All these chemical groups are common when compared to chemical profile of best known medicinal mushrooms and are also responsible for their biological activity [20,24,25].

In the next step, identification of individual compounds from specific groups, including indoles, phenolic acid, and sterols, was performed by High-Performance Liquid Chromatography. Indole compounds present in mushrooms can be divided into two groups: tryptamine derivatives with hallucinogenic properties and bioactive indole derivatives without hallucinogenic properties [26]. Among the non-hallucinogenic indole derivatives indicated in HA, L-tryptophan and 5-hydroxy-L-tryptophan have soporific properties and are supportive in depression treatment. They are also precursors of serotonin and melatonin—endogenous substances responsible for regulating the circadian cycle of our body. Serotonin has also proven antioxidant and anticancer properties. Moreover, it can be used to treat depression and migraine [27,28]. In the fruiting bodies, three indole compounds have been quantified: 5-hydroxy-L-tryptophan, L-tryptophan, and 6-methyl-D,L-tryptophan (Table 1 and Figure S2). Besides, small amounts of melatonin were also qualitatively determined in the extract. From the indole compounds, 5-hydroxy-L-tryptophan (39.11 mg/100 g d.w.) was determined in the highest amounts in fruiting bodies. Significant amounts of L-tryptophan have also been obtained (34.95 mg/100 g d.w).

Analyses of several dozens of species of cultivated and wild growing mushrooms of the Ascomycota and Basidiomycota divisions, i.e., *Agaricus bisporus, Aleuria aurantia, Boletus edulis, Cantharellus cibarius, Cortinarius varius, Cortinarius collinitus Fr., Gymnopilus penetrans, Gymnopilus spectabilis, Lactarius deliciosus, Leccinum scabrum, Lentinula edodes, Mycena pura, Mycena rosea, Paneolus spinctrinus, Pleurotus ostreatus, Russula ochroleuca*, have demonstrated the presence of numerous non-hallucinogenic indole derivatives [29,30]. Analysis of the indole compounds content in *Phellinus*, a genus of mushrooms growing on wood, demonstrated the presence of three indole metabolites including L-tryptophan. The content of L-tryptophan was 8.32 mg/100 g d.w., which is less compared to extract from wild growing HA [31]. In ethanolic extracts from mycelial cultures of the edible *Tricholoma equestre* and *Imleria badia*, the content of 8 indole derivatives has also been determined. Three common metabolites in fruiting bodies of both species were found, L-tryptophan, tryptamine, and serotonin, while in the biomass from in vitro cultures 2 compounds, L-tryptophan and tryptamine. The relatively high content of L-tryptophan, serotonin, and tryptamine was noticeable [32]. These results are similar to extract from HA because high amounts of indole compounds have been quantified in fruiting bodies. Analysis of indole compound content in other fruiting bodies and mycelial cultures of edible mushroom *C. cibarius* demonstrated the considerable content of 5-hydroxy-L-tryptophan (similar amounts to 12.52 mg/100 g d.w.) [29]. Furthermore, in this case, the HA species proved to be a better source of 5-hydroxy-L-tryptophan.

It is established that the most commonly occurring phenolic acids in sporocarps of Basidiomycota are protocatechuic, gallic, *p*-hydroxybenzoic, gentisic, caffeic, syringic, and vanillic acids [33]. Phenolic acids determined in the fruiting bodies of HA were protocatechuic acid in the amount of 2.24 mg/100 g d.w. and gentisic acid in the amount of 76.47 mg/100 g d.w. (Table 1). Both of them are important for the survival of fungal organisms, as evidenced by the various biogenetic pathways leading to their formation. These compounds play an important role against fungal, parasites, and microorganisms invasion. Thus, their insecticidal, anti-bacterial, and anti-fungal properties are known. It should be emphasized that the aforementioned properties are directly associated with their antioxidant activity and affect the ability to help the endogenous defense system and regulation of host microbiota. These immunomodulatory properties are crucial during the prevention and treatment of different pathologies, including cancer [34]. Moreover, these compounds play an important role in the case of arboreal species. During decomposition processes of wood where arboreal mushrooms exist, catalyzed by several enzymes, i.e., ligninase, hemicellulose, cellulose, oxidase, and peroxidase (Lignin peroxidase–LIP, Mn-peroxidase–MnP), there is an increased production of free oxygen radicals that induce the process of fatty acids autooxidation. Probably to avoid oxidative damage, polyporoid fungi, during the evolution process, have developed the ability to synthesize antioxidants and their main representatives are phenolic acids [35]. Gentisic acid is a well-known and used anti-inflammatory and analgesic drug [36]. It is obtained as a result of Kolbe reaction from hydroquinone, but it also can be obtained biotechnologically by the strains *Penicillium patulum* and *Polyporus tumulosus*. The protocatechuic acid (3,4-dihydroxybenzoic acid) is a natural polyphenol found in many edible and medicinal plants and fungi. Studies conducted in recent years indicate that it can be used in the prevention of cardiovascular disease and cancer. The mechanism of action of protocatechuic acid is based on its antioxidant properties, i.e., inhibiting the generation of free radicals, the ability to scavenge them, and increasing the catalytic activity of endogenous enzymes involved in the neutralization of free radicals. The significant effect of protocatechuic acid on the enzymes involved in the first and second stages of biotransformation of some carcinogens should also be highlighted. Their mode of action probably based on possibly blocking specific binding sites of metabolized carcinogens with the DNA molecule, which consequently prevents the formation of adducts that can cause mutations and cancer transformations. However, the other aspects of the chemopreventive activity of protocatechuic acid, i.e., influence on the activity of both inducible isoenzyme of cyclooxygenase and nitric oxide synthase or cell cycle regulating proteins, are not fully proven. Nevertheless, they can be helpful during the explanation of observed anticancer efficacy.

Among the determined sterols, ergosterol and ergosterol peroxide were identified (in quantities 9.48 and 23.85 mg/100 g d.w. of extract, respectively) (Table 1 and Figure S3). Sterols are common ingredients of fruiting bodies of most Basidiomycota representatives. The most common is ergosterol. It has been proven that ergosterol and its peroxide are essential for the proper development of hyphae of higher fungi. Ergosterol (provitamin D_2_) is one of the main components of fungal cell membranes. It is also a precursor of cortisol, an adrenal hormone with anti-inflammatory activity, exerting anticancer and immunostimulatory effects [37]. Moreover, numerous studies have shown that ergosterol and its peroxidation products (ergosterol peroxide) display a therapeutic effect in the reduction of pain associated with inflammation, reduce the incidence of cardiovascular disease, and inhibit the action of the enzyme cyclooxygenase (COX) [38]. Additionally, ergosterol peroxide has a broad spectrum of biological properties, such as antioxidant, anticancer, anti-inflammatory, or antimicrobial activity [39]. Besides, it is believed that ergosterol peroxide is present as an intermediate in the H_2_O_2_-dependent enzymatic oxidation reaction in the steroid biosynthetic pathway or as a detoxification product of reactive oxygen species. Its content in cells depends on many factors, including from the level of reactive oxygen species and the individual relationship between the formation of ergosterol peroxide and its re-conversion to ergosterol. It was also indicated that the presence of substances with antioxidant properties in biomass may affect the ratio of ergosterol to ergosterol peroxide [39].

According to results from F-AAS analysis in the mushroom material, four bioelements important for human health (Cu, Fe, Mg, and Zn) were identified. Magnesium was determined in the highest amount (186.66 mg/100 g d.w.) (Table 1). In addition, it is proved that the fruiting bodies of HA are a good source of Fe (14.21 mg/100 g d.w.) and slightly worse source of Zn (4.20 mg/100 g d.w.). Cu was found in the lowest amounts. The amount of Zn detected in another mushroom *I. badia* and its mycelial cultures was found to be around 12.13 mg/100 g d.w., which is much higher quantity compared to HA content. It shows the specific properties of mycelial cultures and mushrooms and their ability to absorb specific bioelements from the medium on which they grow [40]. In *T. equestre* fruiting bodies, content of bioelements such as Mg (28.6 mg/100 g d.w.), Cu (4.9 mg/100 g d.w.), Fe (98 mg/100 g d.w.), and Zn (17 mg/100 g d.w.) were a bit different also. Only in the case of Mg, higher amounts were determined in fruiting bodies of HA compared to the *T. equestre* species [41].

Taking together, detailed physicochemical analysis of the crude methanolic extract of HA performed in this study might explain the observed anticancer activity. Since there is evidence of the bioactive potential of medical fungi, the chemical and biological importance of these is thus enhanced. According to their pleiotropic properties including immunomodulatory, anticancer, and antimicrobial properties, mushrooms might be used directly in diet and promote health or as adjuvant therapy, taking advantage of the additive and synergistic effects of all the present bioactive compounds.

### 2.3. Hemocompatibility and Cytotoxic Activity of HA Extract at In Vitro and In Vivo Level

Tumor diseases are one of the main causes of death worldwide. Subsequently, cancer has become the most intense field in life science, thus it is one of the main conditions where medicinal mushrooms have been used. Few extracts got a license as adjuvant nutrition in cancer therapy [42]. Wide range of immunomodulatory effects that make medicinal mushrooms ideal for support in cancer cases comprises the capability to facilitate more effective immune response to cancer cells, ability to increase apoptosis of tumor cell and to impede tumor growth and metastasis, and ultimately the possibility to increase the efficacy with side-effects reduction of conventional therapy [43,44,45]. Nowadays, it is expected that medicinal mushrooms and their synthetic derivatives would play a more significant role in the development of innovative products in cancer prevention. Current anticancer drugs have been shown to exert numerous side effects. This underlines the necessity of the implementation of novel and less toxic agents, such as from natural products.

With regard to all of this, our research was performed to establish a novel approach to HA. This basidiomycete fungus is a very important, necrotrophic pathogen on Pinaceae; however, its potential antitumor effect has not been described yet.

#### 2.3.1. High Compatibility and Restriction of CRC Cell Viability after Exposure to HA Extract

**Figure 2:**
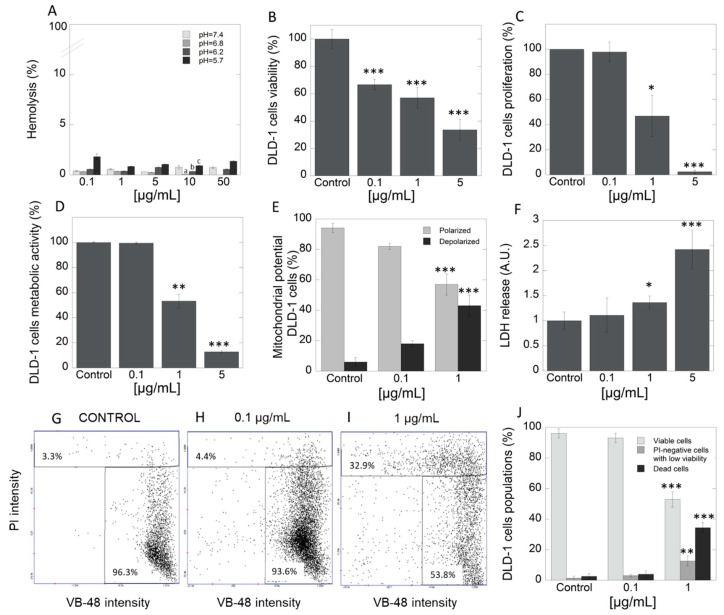
Hemocompatibility and cytotoxicity of *Heterobasidion annosum* (HA) extract against DLD-1 cells. Panel **A** shows high compatibility of HA extract against representative of host cells–RBC cells. The ability to decrease viability (panel **B**) and proliferation (panel **C**) of DLD-1 colorectal cancer cells after treatment with HA extract at different concentration range 0.1–5 µg/mL. Pleiotropic mode of action of HA extract—reduction of metabolic activity (**D**), increasing number of depolarized cells (**E**), and leakage of LDH from the treated cells (**F**). The distribution of thiol levels in DLD-1 cells after treatment by HA extract- fluorescence intensity scatters (**G**-control, **H**-0.1 µg/mL and **I**-1 µg/mL) and column plot (**J**). PI—propidium iodide, VB-48—VitaBright-48 iodide; Statistical significance: (pH = 7.4) vs. (pH = 6.8) ^a^ *p* < 0.01; (pH = 6.8) vs. (pH = 6.2) ^b^ *p* < 0.01; (pH = 6.8) vs. (pH = 5.4) ^c^ *p* < 0.01 (panel **A**); * *p* < 0.05 vs. control, ** *p* < 0.01 vs. control; *** *p* < 0.001 vs. control.

The study was performed with the use of HA extract, in which concentrations were selected based on multiple assays using many concentrations due to lack of such data in literature. In the first step, hemocompatibility of tested extract was evaluated. Data in Figure 2A indicate that HA extract does not affect membrane permeability of red blood cells (RBCs) at a concentration range of 1–50 μg/mL. Determination of hemolytic activity showed that tested extract applied at different concentration range exerts high hemocompatibility against the representative of host cells, RBCs. In addition, in our study, we also assessed the pH-dependent hemolysis assay that is generally engaged as a model for screening pharmaceutical agents designed for intracellular delivery of biologic drugs. Results indicate that there was no hemolytic activity of HA extract at any concentration and different pH. However, results indicated the increasing hemolytic potential in tested concentration as the pH decreases. This could suggest that, in the tested extract, some compounds with endosomolitic potential might exist. The above results are important as they signalize high level of safety in case of potential intravenous application of the product. DLD-1 is an epithelial, adherent cell line derived from a colorectal adenocarcinoma (Dukes type C). This cell line possesses unique features, including high tumorigenicity, and is appropriate to in vivo evaluation in a xenograft model in nude mice. Moreover, it is characterized by positive expression of different genes, such as *myc*, *myb*, *ras*, *fos*, *sis*, *p53*, *miR-21*, which determine their resistance for treatment by classical chemotherapy [46]. Viability of DLD-1 cell line, which is representative of CRC, after exposure to three different concentrations of HA extract (0.1, 1 and 5 μg/mL), presents Figure 2B as a percent of control. For this purpose, neutral red assay was employed. Significant decrease in viability of DLD-1 cells in concentration-dependent manner was noted. To support the observed anticancer potential, significant suppression of cell proliferation was indicated in DLD-1 cell line at concentrations 1 and 5 µg/mL of HA extract (Figure 2C). For this purpose, [3H]-thymidine incorporation test was engaged. These results clearly show that even micro concentrations of tested extract exhibit high cytotoxic activity. Unfortunately, there is very little data in the literature to discuss; however, due to the rising evidence about the development of cancer cells resistance, increasing interest in searching for alternative therapeutic options has been noted. Tomasi et al. screened extracts of 58 mushroom species for their cytotoxic activities against two murine cancer cell lines, L1210—lymphocytic leukemia (ATCC CCL 219) and 3LL—Lewis lung carcinoma (CRL-1642). Among tested methanol extracts, HA was also included. Nonetheless, the authors claimed that extract from HA was inactive and exhibited IC50 > 100 µg/mL against both cell lines [47]. According to the standards of the National Cancer Institute (NCI), extract may be recognized as significantly cytotoxic with IC50 < 20 µg/mL [48].

#### 2.3.2. Exposition to HA Extract Induces Apoptosis via the Complex Mode of Action

Currently, anticancer drugs available on the market have numerous adverse effects. In view of the above, the search for new strategies such as the application of extract from natural sources where interaction between multiple compounds with different modes of action takes place, is the current focus of research. To evaluate the HA extract’s mode of action, which is responsible for observed cytotoxic and antiproliferative efficacy, a number of techniques including study of cells metabolic activity, mitochondrial transmembrane potential, lactate dehydrogenase (LDH) release assay, distribution of thiol levels as well as expression of specific apoptotic-associated proteins in DLD-1 cell culture were carried out. In the first step, the impact of the extract on cells’ metabolic activity has been performed. Figure 2D shows that the addition of HA extract caused depletion of cells metabolism in concentration-dependent manner. Importantly, a significant reduction of metabolic activity (over 8-fold) was observed in cells with HA extract in a concentration of 5 µg/mL, compared to control. Results from evaluation of mitochondrial transmembrane potential showed the highest percentage of cells with properly polarized mitochondria in the control group (94.2%) (Figure 2E). An increase in the percentage of cells with depolarized mitochondrial membrane was observed after administration of 0.1 μg/mL and 1 μg/mL of HA extract, 17.9% and 42.6%, respectively. In turn, as presented in Figure 2F, the addition of tested extract at the concentration range 0.1–5 μg/mL strongly increased the release of LDH from treated cancer cells. This is a result of the disruption of the plasma membrane and leakage of cytoplasmatic content from the treated cells. The abovementioned HA extract activity might suggest that, in the mixture of active agents, membrane-active agents might be presented. This provides the non-specific mode of action, against which the creation of the resistance phenomena is strongly restricted. This finding confirms the results obtained in the vitality assay (Figure 2G–I). About 96.3% of healthy cells and 3.3% of apoptotic cells were observed in the control group. Addition of HA extract (0.1 and 1 μg/mL) evoked a growth in rate of apoptotic cells (4.4% and 32.9%, respectively). Taking together, performed complex studies in DLD-1 cell line revealed increasing percentage of apoptotic cells with increscent concentrations of tested extract. It is associated with the fact that the decrease in cellular glutathione (GSH) concentration is an early hallmark in the progression of cell death in response to apoptotic stimuli.

**Figure 3:**
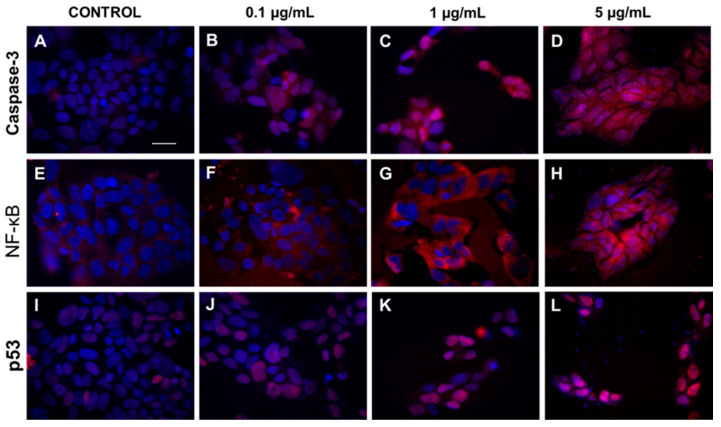
The cytoplasmatic and nucleus level of caspase-3, NF-κB, and p53 in DLD-1 cell line after HA extract application. Panels (**A**–**D**) show expression of caspase 3 after treatment of DLD-1 by different concentrations of HA extract (0.1–5 µg/mL). Increasing expression of NF-κB and p53 protein after addition of HA extract at different concentration range 0.1–5 µg/mL are showed at panels (**E**–**H**) and (**I**–**L**), respectively. Magnification 400×, Scale bar = 20 μm.

To prove the obtained results, immunofluorescence microscopy was used. Results presented in Figure 3 showed increased expression of caspase-3 and translocation of caspase-3 and p53 protein from cytoplasm to cell nucleus in cells exposed to increasing concentrations of HA extract (0.1, 1, and 5 µg/mL).

It is established that antitumor effect might be associated with different pathway including inhibition of proliferation, induction of cell cycle arrest, increased apoptosis, or regulation of signal transduction pathways [49]. In agreement with our studies, Youn et al. showed that water extract from *Inonotus obliquus* could significantly inhibit the viability and proliferation, as well as induce apoptosis in human hepatoma cells (HepG2) [50]. Other authors treated HT-29 human colon cancer cells with 1.0 mg/mL of *I. obliquus* water extract. Maximum inhibitory effect that they could observe was 56%, with concomitant Bcl-2 downregulation and Bax and caspase-3 upregulation. Ultimately, this led to inducing apoptosis of cancer cells. Others also found a decrease in Bcl-2 expression with a simultaneous increase in caspase-3 and Bax or p53 expression. However, this was observed in different extract concentrations and cancer cell lines [51,52,53]. The above results, though applied to different mushrooms, are in agreement with our findings concerning caspase-3 and p53 protein. We showed increased expression of caspase-3 and translocation of caspase-3 and p53 protein from cytoplasm to cell nucleus in cells exposed to increasing concentrations of HA extract. Due to lack of data, this outcome could not be compared with similar results featuring HA extract.

#### 2.3.3. Effects of HA Extract in Xenograft CRC Model

**Figure 4:**
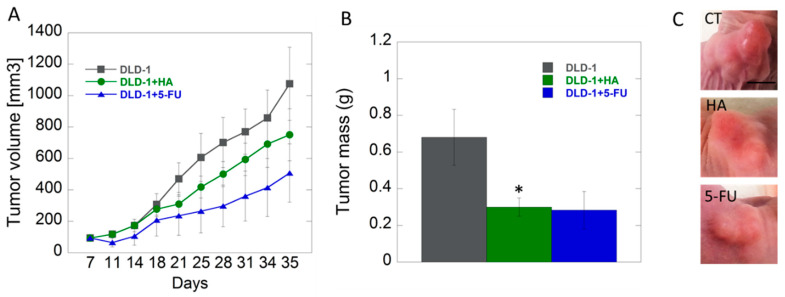
Analysis of tumor volume (panel **A**) and tumor mass (panel **B**) changes in study groups (control–DLD-1, HA-1 mg/kg body weight, DLD-1+5-FU); example images of tumors (**C**), scale bar = 0.5 cm * *p* < 0.05 vs. control.

The in vitro results were confirmed by our subsequent xenografted tumor model in vivo. The studies performed on animals with inoculated DLD-1 colorectal cancer cells showed that growth of tumors in studied groups was approximate up to 18th day, and the differences, however not statistically significant, became noticeable in the second half of experiment. We noticed lower tendency of tumor growth in the group of mice receiving HA extract compared to the control group. The smallest tumor growth was noted in the group of animals receiving 5-fluorouracil (5-FU), as a positive control, intraperitoneally (Figure 4). 5-FU belongs to the group of anti-metabolites that interferes with the synthesis and stability of DNA and RNA of nucleic acids. Its mode of action causes the conversion of fluorodeoxyuridine monophosphate (FdUMP), which forms a stable complex with thymidylate synthase (TS), and thus inhibits deoxythymidine mono-phosphate (dTMP) production, which is essential for DNA replication, its repair, and depletion, therefore, causing cytotoxicity [54].

There are no data involving HA in terms of its biological activity and thus our study is innovative. To date, the influence of medicinal mushrooms on the growth of colon cancer is described in limited number of papers. Li et al. tested *Hericium erinaceus* extracts against HT-29 colon cancer, HepG2 and Huh-7 liver cancer cells, and NCI-87 gastric cancer cells in vitro and tumor xenografts bearing in SCID mice in vivo [55]. Their results showed that extracts are active against cancer cells in vitro and also more effective and less toxic compared to 5-FU in in vivo tumor models [55]. In another study by Kang et al., beneficial effect of active compounds from *Inonotus obliquus* was presented on Azoxymethane/Dextran sulfate sodium colon cancer model [56]. Anticancer effects of various medicinal mushrooms are presented on other tumor models. Wang et al. showed apoptotic potential of *Phellinus igniarius* extract (TPI) on gastric cancer cells. Published results showed that the application of TPI markedly inhibited the viability of human cancer lines A549, HepG-2, Hela, AGS, and SGC-7901 in vitro, which was consistent with in vivo results performed on xenograft mouse model. After two weeks of TPI treatment of nude mice bearing SGC-7901 cells, the tumor size and weight in the groups that received TPI at doses 400 and 800 mg/kg were significantly reduced compared to the control group [57]. Antitumor activity of polysaccharides from *Inonotus obliquus* was noted by Fan et al. [58]. The growth of gastric carcinoma in in vivo was significantly inhibited by tested polysaccharides compared to control [58]. Won et al. presented that polysaccharides isolated from fruiting bodies of *Inonotus obliquus* reduced melanoma tumor growth in tumor-bearing mice [59].

Additionally, only a few studies focus on the synergistic effects of mushrooms with 5-FU. Yang L. et al. examined the activity of 5-FU in hepatocellular carcinoma (HCC) [60]. Due to the significant resistance of tumor cells to standard chemotherapy regimens, researchers added to 5-FU anagrafolide (ANDRO), a bicyclic diterpenoid isolated from *Andrographis paniculata*. Synergistic induction of apoptosis was observed under the influence of 5-FU + ANDRO polytherapy, which was confirmed by increased activity of caspase-8, p53, and significant changes in Bax conformation in cancer cells [60]. Opattova et al. analyzed simultaneous effect of *Ganoderma lucidum* (GLC) with 5-FU in mice xenograft model of colorectal cancer (CT26.WT cells) [61]. GLC positively influenced the cytotoxic effect of 5-FU on tumor size, and better survival was observed in the group of mice treated with GLC and 5-FU together [61].

A tendency to decrease tumor size after HA administration observed in the present study suggests that the experiments should be extended. Our goal is to develop a dose of HA to achieve a reduction in the growth of colorectal tumors to the values obtained after 5-FU administration. Similar to above-cited papers, scheme of synergistic action of HA and 5-FU is under investigation.

#### 2.3.4. HA Extract Applied in Doses up to 2000-Fold Effective Dose Caused Mild or Moderate Acute Toxicity

In the evaluation of acute oral toxicity, HA extract in doses of 175, 560, 1792, and 2000 mg/kg body weight (b.w.) was used. Some toxicity up to 24 h after administration has been noted. The majority of these changes were mild or moderate in intensity (e.g., depression, hypopnea, gait disturbance, hypersalivation) and resolved from 6 to 10 h or even sooner. Five animals were sacrificed on the first day or later, however, 2 of them were from the control group. One mouse which received an extract in concentration 560 mg/kg b.w. and two after 1792 mg/kg b.w., and also 1 from the control group, survived 14 days of the experiment without any toxic effects. Mice that were sacrificed due to toxicity or survived 14 days of the experiment have undergone macroscopic and microscopic observations. The most frequent changes included organ hyperemia and petechiae.

**Figure 5:**
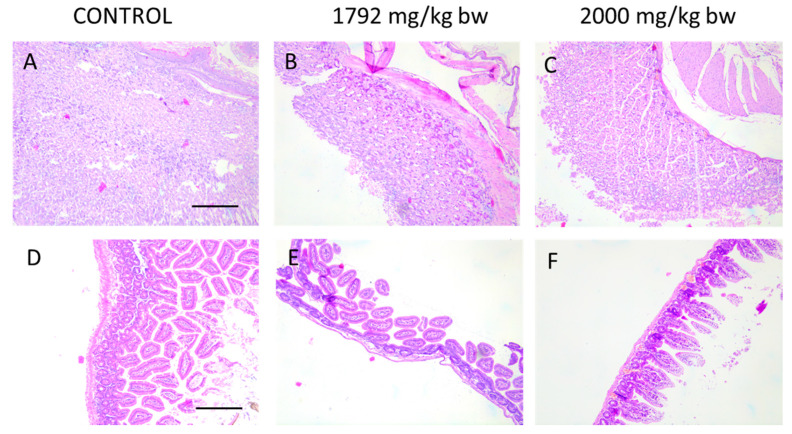
Histological images of stomach (**A**–**C**) and intestine (**D**–**F**) showing no pathological changes in animal receiving the highest doses of HA extract in acute toxicity study. Magnification 100×.

Due to oral administration of HA extract, macroscopic and microscopic analysis of the gastrointestinal tract have been performed. As microscopic images (Figure 5) of intestinal and stomach mucosa of animals from acute toxicity studies show, there are no pathological changes after treatment of mice with the highest doses of HA extract (1792 and 2000 mg/kg b.w.).

Therefore, we cannot say if there was a toxic effect of HA extract or DMSO, especially when there was no correlation between extract dose and toxic effect. On the basis of the obtained results, it is not possible to establish any clear conclusions or to determine the maximum tolerated dose (MTD) of HA extract. It must be noted that concentrations used in the acute toxicity experiment exceeded up to 2000-fold effective dose used in the experiment, which suggests that even if the results do not point specific toxic doses, in therapeutic doses, none of the toxic symptoms were observed.

As a conclusion, it should be emphasized that the obtained results suggest that precise dose selection is obligate, especially concerning the fact that HA is not classified as an edible mushroom.

## 3. Materials and Methods

### 3.1. Biological Material and Morphological Identification

The fruiting bodies of HA were collected using the route method in September 2013 in the Białowieża Forest (Hajnówka Forest District, Hajnówka, Poland) from the trunks of the infected *Pinus sylvestris* L. Photographs were taken in natural conditions, documenting the general habit and features of the fruiting bodies that may have blurred during drying. Microscopic examination of fruiting bodies was used for systematic identification. The taxonomic identification of HA fruiting bodies was made by Marek Wolkowycki with OPTA TECH (Opta-Tech, Warsaw, Poland) binocular magnifier under 10–25× magnification. Tubular and context microscopic preparations were also made. To observe the microscopic features, the OPTA TECH LAB-40 light microscope with variable phase contrast was used. Preparations from fresh specimens were made in water, and from dry samples with 3–5% KOH. For documentation purposes, dried fragments of the fruiting bodies were placed in the Fungarium of the Institute of Forest Sciences in Hajnówka.

### 3.2. PCR Amplification and Sequencing of the Fungal ITS Region

DNA extraction procedure was performed according to the method described by Borges et al. [62]. The purity and quantity of the DNA samples were evaluated using an ND-1000 spectrophotometer (Thermo Scientific, Waltham, MA, USA). PCRs were performed using DreamTaq Green PCR Master Mix (Thermo Scientific, Waltham, MA, USA) in a T-Personal thermal cycler (Biometra, Göttingen, Germany). To confirm the genetic identity of the fungus, the ribosomal RNA gene fragment was amplified using the universal primers ITS1 (TCCGTAGGTGAACCTGCGG) and ITS4 (TCCTCCGCTTATTGATATGC) as described previously. The amplified region was analyzed by direct sequencing of the PCR products. Automatic sequencing was performed using a BigDye™ Terminator Cycle Sequencing Kit and ABI PRISM 310/3730 XL sequencers (Applied Biosystem, Foster City, CA, USA). Data from ITS sequencing was analyzed with ChromasPro v.1.6 (Technelysium Pty Ltd., South Brisbane, Australia) and Lasergene v.11.0 software (DNASTAR, Inc, Madison, WI, USA). Database searches were performed with the BLAST program at the National Centre for Biotechnology Information (Bethesda, Rockville, MD, USA) [63].

### 3.3. Preparation of HA Extract

Fresh fruiting bodies of HA were cut and weight at 100 g. Raw material was transferred to screw-cap bottles with 200 mL methanol. Extraction was repeated three times for each sample. Then, extracts were combined and filtered through the paper filter. Methanol was evaporated and dry residues were used for biological activity research and determination of bioactive substances content.

For in vitro and in vivo experiments, HA extract was dissolved in pure DMSO first and diluted with sterile saline (0.9% NaCl) with the final DMSO concentration at 2%.

### 3.4. Physicochemical Analysis of HA Extract

#### 3.4.1. FTIR Spectroscopy Analysis.

The analysis was carried out using dry extract. FTIR spectroscopy was performed with a FTIR spectrometer Nicolet 8700A coupled to the FT Raman Nicolet NXR spectrometer module by Thermo Scientific, Waltham, MA USA in the wavelength range 4000–400 cm^−1^.

#### 3.4.2. Gas Chromatography–Mass Spectrometry Analysis

The samples for GC/MS analysis were prepared as follows: 10 mg of methanolic HA extract was diluted with 1 mL of pyridine and 200 µL N,O-bis(trimethylsilyl)-trifluoroacetamide (BSTFA) was added. The mixture was heated for 30 min at 60 °C. The silylated samples were separated on an Agilent 7890A gas chromatograph equipped with an Agilent 5975C mass selective detector. The injection of 1 µL of sample was done using an Agilent 7693A autosampler. The separation was performed on an HP-5MS (30 m × 0.25 mm × 0.25 µm film thickness) fused silica column at a helium flow rate 1 mL/min. The injector worked in a split (1:50) mode. The injector temperature was 300 °C. The initial column temperature was 50 °C, rising to 320 °C at 3 °C/min; the final temperature was held for 10 min. The ion source temperature was 230 °C and quadrupole temperature was 150 °C. The electron ionization mass spectrometry (EIMS) were obtained at 70 eV ionization energy. Detection was performed in a full scan mode from 41 to 600 a.m.u. After integration, the fraction of each component in the total ion current (% of TIC) was calculated. The retention indices of analytes were determined using alkanes retention times.

To identify components, the mass spectral data and the calculated retention indices were used. Mass spectrometric identification was carried out with an automatic system of GC-MS data processing supplied by NIST—The National Institute of Standards and Technology and book “Identification of Biologically and Environmentally Significant Organic Compounds Mass Spectra and Retention Indices Library of Trimethylsilyl Derivatives”. The retention indices of the registered compounds were compared with those presented in the NIST database and abovementioned book.

#### 3.4.3. High-Performance Liquid Chromatography

##### (a) Analysis of Indole Compounds

The methanolic extracts were quantitatively dissolved in 1.5 mL of solvent system (methanol/water/ammonium acetate 15:14:1 *v*/*v*/*v*) and subjected to isocratic separation by RP-HPLC, employing a Hitachi HPLC (Merck, Tokyo, Japan) equipped with a type L-7100 pump. The Purospher^®^ RP-18 (4 mm × 200 mm, 5 µm) column was kept at 25°C and the UV detector operated at λ = 280 nm. The liquid phase used was a mixture of methanol/water/ammonium acetate (15:14:1 *v*/*v*/*v*) at a flow rate of 1 mL/min. The quantitative analyses of indole compounds were performed using a calibration curve with the assumption of the linear size of the area under the peak and the concentration of the reference standard (Sigma–Aldrich, St. Louis, MO, USA).

##### (b) Analysis of Phenolic Compounds

Methanolic extracts were quantitatively dissolved in methanol (1.5 mL) and filtered through a Millipore Millex–GP, 0.22 µm. The resultant extracts were analyzed for their contents of phenolic acids by the RP-HPLC method. These analyses were carried out according to the procedure developed by Muszyńska et al. 2016, with some modifications [64]. HPLC analyses were conducted using an HPLC VWR Hitachi-Merck apparatus: L-2200 autosampler, L-2130 pump, RP-18e LiChrospher (4 mm × 250 mm, 5µm) column thermostated at 25 °C, L-2350 column oven, L-2455 diode array detector at the UV range 200–400 nm. The mobile phase consisted of solvent A: methanol/0.5% acetic acid 1:4 (*v*/*v*), and solvent B: methanol. The gradient was as follows: 100:0 for 0–25 min; 70:30 for 35 min; 50:50 for 45 min; 0:100 for 50–55 min; 100:0 for 57–67 min. The comparison of UV spectra and retention times with standard compounds enabled the identification of phenolic acids present in analysis samples. The quantitative analysis of free phenolic acids was performed with the use of a calibration curve with the assumption of the linear size of the area under the peak and the concentration of the reference standard (Sigma–Aldrich, St. Louis, MO, USA).

##### (c) Extraction and Analysis of Sterol

Mushroom methanolic extract was mixed with a mixture of methanol/dichloromethane 75:25 (*v*/*v*). The mixture was sonicated at 40 kHz for 10 min. After 2 h, the extract was centrifuged at 12,000 rpm for 5 min and decanted. The extraction procedure was repeated twice and obtained extracts were mixed and evaporated under reduced pressure. The filtered sample (Millipore PTFE membrane 0.45 µm) was injected (20 µL) in the HPLC column. HPLC analyses were conducted using HPLC VWR Hitachi liquid chromatograph (Merck, Darmstadt, Germany) described above. The HPLC method was followed according to the procedure developed by Yuan et al. with own modifications of gradients procedure [65]. The mobile phase consisted of solvent A: methanol/water 80:20 (*v*/*v*), and solvent B: methanol/dichloromethane 75:25 (*v*/*v*). A gradient program was as follows: 0–10 min, 80:20% B; 10–35 min, 40–60% B; 35–50 min, 0–100% B; 50–55 min, 80–20% B; with a hold time of 15 min, at 30 °C. The flow rate was 1.0 mL/min. The chromatographic peaks were recorded at a wavelength of 280 nm. Sterol standards were purchased from Fluka (Chemie AG, Buchs, Switzerland).

#### 3.4.4. F-AAS Analysis of Selected Bioelements

The dried mushroom fruiting bodies were weighed (0.2 g) and then mineralized in a closed microwave system Magnum II (Ertec, Wrocław, Poland). Mineralization was carried out in three stages of 10 min each using Suprapur^®^ HNO_3_ (65%) and H_2_O_2_ (30%), at a power of 70% and 100%, respectively, maintaining the temperature of the device at 290 °C. After mineralization, the solutions were transferred to quartz evaporators and evaporated on a heating plate at 180 °C to remove excess reagents and water. The residue obtained after evaporation was quantitatively transferred to 10 mL volumetric flasks with four-times-distilled water. To analyze the composition of bioelements such as Cu, Fe, Zn, and Mg, the standard solutions were used. Bioelements were analyzed using the atomic absorption spectroscopy (AAS) method with flame atomization (AAS iCE3300 Thermo Scientific™ spectrophotometer, Cambridge, UK).

### 3.5. In Vitro Evaluation of HA Extract

#### 3.5.1. Hemolytic Activity

To evaluate the hemocompatibility of fungal extract, a hemolysis assay was performed. In brief, human red blood cells (RBCs) were suspended in phosphate-buffered saline (PBS pH = 7.4) (hematocrit ~5%), and then tested extract was added at a concentration ranging from 0 to 50 μg/mL. In another set of experiments, using the same concentration range, pH-dependent hemolysis, which is a model of endosomolytic drug delivery agents, was performed. RBCs were incubated with tested extract at different pH 5.6, 6.2, and 6.8. In all conditions, RBCs were incubated with tested agents for 1 h at 37 °C. Relative hemoglobin concentration in supernatants after centrifugation at 2500× *g* was monitored by measuring optical absorbance at 540 nm. 100% hemolysis was taken from samples, where 1% Triton X-100 was added in order to disrupt all cell membrane [66].

The hemolytic activity of the tested agents was evaluated in blood samples from adult healthy volunteers under IRB approval: R-I-002/245/2019. This study was approved by the Institutional Review Board (IRB) of The Medical University of Bialystok, Bialystok, Poland. All subjects provided informed written consent and collected samples were anonymous.

#### 3.5.2. Cell Culture

Studies were performed on colon cancer DLD-1 cell line, purchased from American Type Culture Collection (Manassas, VA, USA). The cells were maintained in Dulbecco’s Modified Eagle Medium (DMEM) with GlutaMax I supplemented with 10% fetal bovine serum (FBS), 50 U/mL penicillin, 50 mg/mL streptomycin at 37 °C in a 5% CO_2_ incubator. Cells were counted in a hemocytometer and cultured at 1 × 10^5^ cells per well in 2 mL of growth medium in 6 well plates or 100 µL of growth medium in 96-well plates (Sarstedt, Nümbrecht, Germany). Cells were used for the assays after they reached confluence. Cells were used in the 8th to 14th passages.

#### 3.5.3. Cell Viability Assay

To determine cytotoxicity of HA extract, different techniques were engaged. In all tested conditions, the cells were maintained as described above. In the first step of studies, measurement of cell viability by neutral red assay has been performed. In brief, after 24 h of incubation with HA extracts (0.1, 1, and 5 µg/mL) neutral red solution was added and incubated for 2 h. Then, the culturing medium was removed, and the cells were fixed for 5 min. Then, fixative solution was discarded, and the dye was solubilized by acidic solution. The absorbance was measured by Biotek microplates reader at 540 nm wavelength [67].

In another set of experiment, evaluation of how HA extract affects the metabolic activity was determined by MTT assay method described by Carmichael et al. [68]. After 24 h of incubation with HA fungus extracts (0.1, 1, and 5 µg/mL), the culturing medium was discarded and the cells were rinsed three times with PBS. Then, the cells were incubated for 20 min with MTT solution (5 mg/mL). Medium was removed from the wells, and the cells were dissolved in 200 µL of DMSO with 20 µL of Sorensen’s buffer (0.1 mol/L glycine with 0.1 mol/L NaCl equilibrated to pH 10.5). The absorbance was recorded with spectrophotometer (Fisher Scientific, Waltham, MA, USA) at wavelength of 570 nm. Values are described as a percent of control.

To evaluate the ability of tested extract to disrupt plasma membrane, the detection of LDH release from treated cells was performed in accordance with a commercially available protocol. Briefly, after 24-h incubation of cells with HA extract, the medium was transferred into 96-well plates, and then 50 µL of Master Reaction mix was added. After 10–15 min of incubation, the absorbance was measured using a Biotek microplates reader at a wavelength of 450 nm [69].

#### 3.5.4. [3H]-Thymidine Incorporation (DNA Biosynthesis Assay)

To examine the effect of HA extracts (0.1, 1, and 5 µg/mL) on cell proliferation, cells were seeded in 24-well plates at 1 × 10^5^ cells/well with 1 mL of growth medium. After 48 h to subconfluent cells, various concentrations of the fungus extract and 0.5 mCi of [3H]-thymidine were added. The incubation was continued for 24 h at 37 °C. Cells were rinsed 3 times with PBS, solubilized with 1 mL of 0.1 mol/L sodium hydroxide containing 1% SDS, then scintillation fluid “Ultima Gold XR” was added and incorporation of the tracer into DNA was measured in scintillation counter.

#### 3.5.5. Mitochondrial Potential Assay

Mitochondrial transmembrane potential (Δψm) was measured using the cationic dye JC-1 (5,5,6,6-tetrachloro-1,1,3,3-tetraethylbenzimidazolcarbocyanine iodide) by advanced image cytometer NucleoCounter^®^ NC-3000™ system (ChemoMetec, Copenhagen, Denmark). This method is based on fluorescent detection of healthy and apoptotic cells. In apoptotic cells, Δψm collapses and JC-1 localizes to the cytosol in its monomeric fluorescent form. Briefly, 1.5 × 10^6^ cells/mL, control or treated with HA extracts (0.1 and 1 µg/mL) for 24 h, were mixed with 200 μg/mL JC-1 (ChemoMetec A/S) and incubated in 37 °C for 10 min. After washing procedures, samples were resuspended in 1 μg/mL of 4′,6-diamidino-2-phenylindole (ChemoMetec A/S) and analyzed with the NucleoCounter^®^ NC-3000™ system.

#### 3.5.6. Vitality Assay: Analysis of the Cellular Thiols (SH)

Vitality assay was estimated using NucleoCounter^®^ NC-3000™ system (ChemoMetec A/S) based on an assessment of the reduced thiol group concentrations, such as reduced glutathione (GSH). Briefly, 4 × 10^9^ cells/mL, control or treated with HA extracts (0.1, and 1 µg/mL) for 24 h, were mixed with VitaBright-48 iodide (ChemoMetec A/S), acridine orange iodide (ChemoMetec A/S), and propidium iodide (ChemoMetec A/S) and immediately analyzed with the NucleoCounter^®^ NC-3000™ system according to the instructions provided by the manufacturer.

#### 3.5.7. Cell Bioimaging

DLD-1 cells were seeded in BD Falcon™ 96-well black/clear bottom tissue culture plates optimized for imaging applications at 10,000 cells per well. After addition of HA extract at concentrations 0.1, 1, and 5 µg/mL, cells were incubated for 24 h at 37 °C in 5% CO_2_. Next, cells were rinsed with PBS and fixed with 3.7% formaldehyde solution at room temperature for 10 min. After fixation, cells were washed three times with PBS and permeabilized with 0.1% Triton X-100 solution at room temperature for 5 min. Then, the cells were washed twice with PBS, and non-specific binding was blocked by adding 3% FBS solution and incubated at room temperature for 30 min. After that, the cells were rinsed, incubated with anti- caspase-9 antibody (concentration of 1:1000), NF-κB antibody (concentration of 1:1000), and p53 antibody (1:1000), respectively, for 1 h at room temperature, washed three times with PBS, and incubated with fluorescent (FITC) anti-rabbit secondary antibody (BD Pharmingen, San Diego, CA, USA) for 60 min in the dark. After washing, nuclei were stained with Hoechst 33342 (2 µg/mL) and analyzed using confocal microscopy imaging. Cells were imaged with a BD Pathway 855 confocal system using a 40× (0.75 NA) objective. Cell populations were analyzed for cytoplasmic/nuclear fluorescence intensity. Images of FITC-labeled cells were acquired using a 488/10 excitation laser and a 515LP emission laser.

### 3.6. In Vivo Evaluation of HA Extract

All procedures were carried out in accordance with the European Directive (2010/63/EU). Handling of animals was performed with the consent of protocols approved by the Local Ethical Committee in Bialystok of the Medical University of Bialystok no 139/2015. Acute oral toxicity of HA extract was carried out according to OECD 425 guidelines. The study protocol was approved by the Local Ethical Committee for Animal Experiments in Olsztyn (No 62/2018).

#### 3.6.1. Xenograft Studies in Nude Mice

Four-week-old NUDE male mice (Cby.Cg.Foxn1<nu>/cmdb) were housed under controlled conditions (21 ± 2 °C, 12 h light ⁄ 12 h dark cycle) with unlimited access to water. DLD-1 cells (2 × 10^7^) were implanted subcutaneously into the flanks of nude mice. After 7 days, mice were randomly divided into three groups (*n* = 6), which were given PBS (control group) or an extract from HA (1 mg/kg b.w.) intragastrically once a day for 28 days or intraperitoneal injection of 5-fluorouracil in a dose of 30 mg/kg b.w. (Sigma-Aldrich, St Louis, MO, USA) on the 21st and 28th day of experiment.

Tumor volume was measured every three days using a digital caliper, and the orthogonal dimensions (length, width) were determined using the following formula: tumor volume = 0.5 × (length × width^2^). After 35 days, mice were sacrificed.

#### 3.6.2. Acute Oral Toxicity of HA Extract

Animals enrolled in the study were female BALB/ccmdb mice of 9–10 weeks. Mean weight of mice at the beginning of experiment was 20.04 ± 0.5 g. Duration of experiment was 14 days.

Extract was dissolved in DMSO and given orally to mice at a single entrance dose of 175 mg/kg/day, which was increased by dose modifying factor 3.2, along with the vehicle control (DMSO) in the volume of 0.2 cm^3^. Mice were given the following doses of extract: 175, 560, 1792, and 2000 mg/kg b.w. (a total volume of 0.2 cm^3^). Higher concentrations were insoluble in the solvent. Animals were observed during the first 12 h for any alteration in the symptoms of mobility, posture, sensitivity to sensory stimulation, the amount of food consumption, and mortality. Mice were weighed every day and observed for fourteen days following treatment. In case of any symptoms suggesting severe pain or other signs of toxicity, mice were humanely sacrificed.

#### 3.6.3. Histopathological analysis

Organs from sacrificed mice were fixed in 10% buffered formalin and embedded in paraffin. From paraffin blocks, 5-μm sections were cut and stained with hematoxylin-eosin (H+E). Analysis of slides was performed using Olympus RSM 400 light microscope.

### 3.7. Statistical Analysis

Statistical analyses were performed using Statistica 13.3 software (StatSoft Inc., Tulsa, OK, USA). The data were analyzed using standard statistical analyses, including one-way analysis of variance (ANOVA) followed by post hoc Duncan’s or Tukey’s test for multiple comparisons. *p*-values less than 0.05 were considered significant.

## 4. Conclusions

In the presented work, for the first time, the cytotoxic effect against CRC of the HA extract was demonstrated in in vivo as well as in vitro conditions. Qualitative analysis of the used mushroom extract showed the presence of many bioactive substances (organic compounds and bioelements) important in controlling many human and animal diseases, i.e., by exerting pleiotropic biological activities for organism such as antioxidant, anti-inflammatory, antitumor, antibacterial, and immunomodulatory activity. The obtained results suggest that the mushroom strain may be an effective source of medicinal substances. In vitro experiments performed on DLD-1 cell line showed significant decrease in cell viability, metabolic activity, and cell proliferation as well as reduced tendency of tumor growth xenograft model of colon cancer. The next step of research should be to expand the number of models to confirm our observations on various cell lines and other scheme of experiments in in vivo models, as well as to explain a specific mechanism of potential anti-colon cancer activity of HA extract as cytotoxic agent or adjuvant anticancer therapy.

## Figures and Tables

**Table 1:** Content of selected organic compounds and bioelements in *Heterebasidion annosum* fruiting bodies based on the results from GC-MS, HPLC, and F-atomic absorption spectroscopy (AAS) analysis. TIC–total ion current; d.w.–dry weight

**Groups of Compounds**	**% of TIC**
Total carbohydrates	6.82
Total sterols	1.82
Total carboxylic acid	1.74
**Indole compounds**	**[mg/100 g d.w.] ± SD**
5-Hydroxy-L-tryptophan	39.1 ± 1.4
L-Tryptophan	34.9 ± 2.4
6-Methyl-D,L-tryptophan	1.1 ± 0.2
Melatonin	*
**Phenolic acids**	**[mg/100 g d.w.] ± SD**
Protocatechuic acid	2.2 ± 0,05
Gentisic acid	76.5 ± 0.7
**Sterol compounds**	**[mg/100 g d.w.]± SD**
Ergosterol	9.5 ± 0.07
Ergosterol peroxide	23.8 ± 0.3
**Bioelements**	**[mg/100 g d.w.] ± SD**
Cu	1.0 ± 0.06
Fe	14.2 ± 1.7
Zn	4.2 ± 0.3
Mg	186.6 ± 4.5

* less than 0.001 mg/100 g dry weight of extract; *n* = 3.

## Supplementary Materials

Supplementary materials can be found at https://www.mdpi.com/1422-0067/21/10/3447/s1.

## Abbreviations

CRC	Colorectal cancer
HA	*Heterobasidion annosum*
RBCs	human red blood cells
GSH	Glutathione
GC/MS	Gas chromatography–Mass spectrometry
HPLC	High-Performance Liquid Chromatography
F-AAS	Atomic Absorption Spectroscopy
FTIR	Fourier-Transform Infrared Spectroscopy
5-FU	5-fluorouracil

## References

[1] Korhonen K., Stenlid J. (1998). Biology of *Heterobasidion annosum*. Heterobasidion Annosum: Biology, Ecology, Impact and Control.

[2] Korhonen K., Capretti P., Karjalainen R., Stenlid J. (1998). Distribution of *Heterobasidion annosum* intersterility groups in Europe. Heterobasidion Annosum: Biology, Ecology, Impact and Control.

[3] Dai Y.C., Yuan H.S., Wei Y.L., Korhonen K. (2006). New records of Heterobasidion parviporum in China. For. Pathol..

[4] Dai Y.C., Vainio E.J., Hantula J., Niemela T., Korhonen K. (2003). Investigations on *Heterobasidion annosum* s. lat. in central and eastern Asia with the aid of mating tests and DNA fingerprinting. For. Pathol..

[5] Otrosina W.J., Garbelotto M. (2010). *Heterobasidion occidentale* sp. nov. and *Heterobasidion irregulare* nom. nov.: A disposition of North American Heterobasidion biological species. Fungal Biol..

[6] Korhonen K. (1978). Intersterility groups of *Heterobasidion annosum*. Commun. Inst. For. Fenn..

[7] Capretti P., Korhonen K., Mugnai L., Romagnoli C. (1990). An Intersterility group of *Heterobasidion annosum* specialized to Abies alba. Eur. J. For. Pathol..

[8] Niemelä T., Korhonen K. (1998). Taxonomy of the genus Heterobasidion. Heterobasidion Annosum: Biology, Ecology, Impact and Control.

[9] Asiegbu F.O., Johansson M., Woodward S., Hüttermann A., Woodward S., Stenlid J., Karjalainen R., Hüttermann A. (1998). Biochemistry of the host–parasite interaction. Heterobasidion annosum: Biology, Ecology, Impact Control.

[10] Asiegbu F.O., Abu S., Stenlid J., Johansson M. (2004). Sequence polymorphism and molecular characterisation of laccase genes of theconifer pathogen *Heterobasidion annosum*. Mycol. Res..

[11] Haars A., Chet I., Hütterman A. (1981). Effect of phenolic compounds and tannin on growth and laccase activity of Fomes annosus. Eur. J. For. Pathol..

[12] Daniel G., Asiegbu F., Johansson M. (1998). The saprotrophic wood-degrading abilities of *Heterobasidium annosum* intersterility groups P and S. Mycol. Res..

[13] Hansson D., Wubshet S., Olson Å., Karlsson M., Staerk D., Broberg A. (2014). Secondary metabolite comparison of the species within the *Heterobasidion annosum* s.l. complex. Phytochemistry.

[14] Bray F., Ferlay J., Soerjomataram I., Siegel R.L., Torre L.A., Jemal A. (2018). Global cancer statistics 2018: GLOBOCAN estimates of incidence and mortality worldwide for 36 cancers in 185 countries. CA Cancer J. Clin..

[15] Moehler M., Göpfert K., Lenz H.J. (2018). Outlook: Immunotherapy in Gastrointestinal Carcinoma—Innovative Strategies. Oncol Res. Treat..

[16] Jo W.S., Hossain M.A., Park S.C. (2014). Toxicological profiles of poisonous, edible, and medicinal mushrooms. Mycobiology.

[17] Lindequist U., Niedermeyer T.H., Jülich W.D. (2005). The pharmacological potential of mushrooms. Evid. Based Complement. Alternat. Med..

[18] Grienke U., Zöll M., Peintner U., Rollinger J.M. (2014). European medicinal polypores—A modern view on traditional uses. J. Ethnopharmacol..

[19] Kladar N.V., Gavarić N.S., Božin B.N. (2016). Ganoderma: Insights into anticancer effects. Eur. J. Cancer Prev..

[20] Glamočlija J., Ćirić A., Nikolić M., Fernandes Â., Barros L., Calhelha R.C., Ferreira I.C., Soković M., van Griensven L.J. (2015). Chemical characterization and biological activity of Chaga (*Inonotus obliquus*), a medicinal “mushroom”. J. Ethnopharmacol..

[21] Patel S., Goyal A. (2012). Recent developments in mushrooms as anti-cancer therapeutics: A review. 3 Biotech.

[22] Simmons E.G. (2010). The International Mycological Association: Its history in brief with summaries of its International Mycological Congresses and diverse international relationships. IMA Fungus.

[23] Garbelotto M., Gonthier P. (2013). Biology, epidemiology, and control of *Heterobasidion species* worldwide. Annu. Rev. Phytopathol..

[24] Su C.H., Lai M.N., Lin C.C., Ng L.T. (2016). Comparative characterization of physicochemical properties and bioactivities of polysaccharides from selected medicinal mushrooms. Appl. Microbiol. Biotechnol..

[25] Chaturvedi V.K., Agarwal S., Gupta K.K., Ramteke P.W., Singh M.P. (2018). Medicinal mushroom: Boon for therapeutic applications. 3 Biotech.

[26] Jiang M.Y., Feng T., Liu J.K. (2011). N-containing compounds of macromycetes. Nat. Prod. Rep..

[27] Keszthelyi D., Troost F.J., Masclee A.A. (2009). Understanding the role of tryptophan and serotonin metabolism in gastrointestinal function. Neurogastroenterol. Motil..

[28] Turner E.H., Loftis J.M., Blackwell A.D. (2006). Serotonin a la carte: Supplementation with the serotonin precursor 5-hydroxytryptophan. Pharmacol. Ther..

[29] Muszyńska B., Sułkowska-Ziaja K., Ekiert H. (2011). Indole compounds in some culinary-medicinal higher basidiomycetes from Poland. Int. J. Med. Mushrooms.

[30] Muszyńska B., Sułkowska-Ziaja K., Ekiert H. (2011). Indole compounds in fruiting bodies of some edible Basidiomycota species. Food Chem..

[31] Sułkowska-Ziaja K., Maślanka A., Szewczyk A., Muszyńska B. (2017). Physiologically Active Compounds in Four Species of *Phellinus*. Nat. Prod. Commun..

[32] Muszyńska B., Sułkowska-Ziaja K., Ekiert H. (2009). Indole compounds in fruiting bodies of some selected Macromycetes species and in their mycelia cultured in vitro. Pharmazie.

[33] Ferreira I.C., Barros L., Abreu R.M. (2009). Antioxidants in wild mushrooms. Curr. Med. Chem..

[34] Pasinetti G.M., Singh R., Westfall S., Herman F., Faith J., Ho L. (2018). The Role of the Gut Microbiota in the Metabolism of Polyphenols as Characterized by Gnotobiotic Mice. J. Alzheimers Dis..

[35] Karaman M., Jovin E., Malbasa R., Matavuly M., Popović M. (2010). Medicinal and edible lignicolous fungi as natural sources of antioxidative and antibacterial agents. Phytother. Res..

[36] Ashidate K., Kawamura M., Mimura D., Tohda H., Miyazaki S., Teramoto T., Yamamoto Y., Hirata Y. (2005). Gentisic acid, an aspirin metabolite, inhibits oxidation of low-density lipoprotein and the formation of cholesterol ester hydroperoxides in human plasma. Eur. J. Pharmacol..

[37] Yokokawa H. (1980). Fatty acid and sterol compositions in mushrooms of ten species of polyporaceae. Phytochemistry.

[38] Zhang Y., Mills G.L., Nair M.G. (2003). Cyclooxygenase inhibitory and antioxidant compounds from the fruiting body of an edible mushroom, Agrocybe aegerita. Phytomedicine.

[39] Krzyczkowski W., Malinowska E., Suchocki P., Kleps J., Olejnik M., Herold F. (2009). Isolation and quantitative determination of ergosterol peroxide in various edible mushroom species. Food Chem..

[40] Kała K., Krakowska A., Gdula-Argasinska J., Opoka W., Muszyńska B. (2019). Assessing the Bioavailability of Zinc and Indole Compounds from Mycelial Cultures of the Bay Mushroom *Imleria badia* (Agaricomycetes) Using In Vitro Models. Int. J. Med. Mushrooms.

[41] Muszyńska B., Kała K., Radović J., Sułkowska-Ziaja K., Krakowska A., Gdula-Argasińska J., Opoka W., Kudaković T. (2018). Study of biological activity of *Tricholoma equestre* fruiting bodies and their safety for human. Eur. Food Res. Technol..

[42] Lemieszek M., Rzeski W. (2012). Anticancer properties of polysaccharides isolated from fungi of the Basidiomycetes class. Contemp. Oncol..

[43] Li Y.H., Niu Y.B., Sun Y., Zhang F., Liu C.X., Fan L., Mei Q.B. (2015). Role of phytochemicals in colorectal cancer prevention. World J. Gastroenterol..

[44] Pandya U., Dhuldhaj U., Sahay N.S. (2019). Bioactive mushroom polysaccharides as antitumor: An overview. Nat. Prod. Res..

[45] Saljoughian M. (2009). Adaptogenic or Medicinal Mushrooms. US Pharm..

[46] Chen J.C., Hsieh Y.Y., Lo H.L., Li A., Chou C.J., Yang P.M. (2019). In Vitro and In Silico Mechanistic Insights into miR-21-5p-Mediated Topoisomerase Drug Resistance in Human Colorectal Cancer Cells. Biomolecules.

[47] Tomasi S., Lohézic-Le Dévéhat F., Sauleau P., Bézivin C., Boustie J. (2004). Cytotoxic activity of methanol extracts from Basidiomycete mushrooms on murine cancer cell lines. Pharmazie.

[48] Cordell G., Kinghorn D., Pezzuto J. (1993). Separation, Structure Elucidation, and Bioassay of Cytotoxic Natural Products.

[49] Swanton C. (2004). Cell-cycle targeted therapies. Lancet Oncol..

[50] Youn M.J., Kim J.K., Park S.Y., Kim Y., Kim S.J., Lee J.S., Chai K.Y., Kim H.J., Cui M.X., So H.S. (2008). Chaga mushroom (*Inonotus obliquus*) induces G0/G1 arrest and apoptosis in human hepatoma HepG2 cells. World J. Gastroenterol..

[51] Song F.Q., Liu Y., Kong X.S., Chang W., Song G. (2013). Progress on understanding the anticancer mechanisms of medicinal mushroom: *Inonotus obliquus*. Asian Pac. J. Cancer Prev..

[52] Ma L., Chen H., Dong P., Lu X. (2013). Anti-inflammatory and anticancer activities of extracts and compounds from the mushroom *Inonotus obliquus*. Food Chem..

[53] Lee H.S., Kim E.J., Kim S.H. (2015). Ethanol extract of Innotus obliquus (*Chaga mushroom*) induces G1 cell cycle arrest in HT-29 human colon cancer cells. Nutr. Res. Pract..

[54] Zhang N., Yin Y., Xu S.J., Chen W.S. (2008). 5-Fluorouracil: Mechanisms of resistance and reversal strategies. Molecules.

[55] Li G., Yu K., Li F., Xu K., Li J., He S., Cao S., Tan G. (2014). Anticancer potential of *Hericium erinaceus* extracts against human gastrointestinal cancers. J. Ethnopharmacol..

[56] Kang J.H., Jang J.E., Mishra S.K., Lee H.J., Nho C.W., Shin D., Jin M., Kim M.K., Choi C., Oh S.H. (2015). Ergosterol peroxide from Chaga mushroom (*Inonotus obliquus*) exhibits anti-cancer activity by down-regulation of the β-catenin pathway in colorectal cancer. J. Ethnopharmacol..

[57] Fan L., Ding S., Ai L., Deng K. (2012). Antitumor and immunomodulatory activity of water-soluble polysaccharide from *Inonotus obliquus*. Carbohydr. Polym..

[58] Won D.P., Lee J.S., Kwon D.S., Lee K.E., Shin W.C., Hong E.K. (2011). Immunostimulating activity by polysaccharides isolated from fruiting body of *Inonotus obliquus*. Mol. Cells..

[59] Yang L., Wu D., Luo K., Wu S., Wu P. (2009). Andrographolide enhances 5-fluorouracil-induced apoptosis via caspase-8-dependent mitochondrial pathway involving p53 participation in hepatocellular carcinoma (SMMC-7721) cells. Cancer Lett..

[60] Wang F.F., Shi C., Yang Y., Fang Y., Sheng L., Li N. (2018). Medicinal mushroom *Phellinus igniarius* induced cell apoptosis in gastric cancer SGC-7901 through a mitochondria-dependent pathway. Biomed. Pharmacother..

[61] Opattova A., Horak J., Vodenkova S., Kostovcikova K., Cumova A., Macinga P., Galanova N., Rejhova A., Vodickova L., Kozics K. (2019). *Ganoderma Lucidum* induces oxidative DNA damage and enhances the effect of 5-Fluorouracil in colorectal cancer in vitro and in vivo. Mutat. Res..

[62] Borges M., Azevedo M., Bonatelli J., Felipe M., Astolfi-Filho S. (1990). A practical method for the preparation of total DNA from filamentous fungi. Fungal Genet. Rep..

[63] Altschul S.F., Madden T.L., Schäffer A.A., Zhang J., Zhang Z., Miller W., Lipman D.J. (1997). Gapped BLAST and PSI-BLAST: A new generation of protein database search programs. Nucleic Acids Res..

[64] Muszyńska B., Łojewski M., Sułkowska-Ziaja K., Szewczyk A., Gdula-Argasińska J., Hałaszuk P. (2016). In vitro cultures of Bacopa monnieri and an analysis of selected groups of biologically active metabolites in their biomass. Pharm. Biol..

[65] Yuan J.P., Kuang H.C., Wang J.H., Liu X. (2008). Evaluation of ergosterol and its esters in the pileus, gill, and stipe tissues of agaric fungi and their relative changes in the comminuted fungal tissues. Appl. Microbiol. Biotechnol..

[66] Evans B.C., Nelson C.E., Yu S.S., Beavers K.R., Kim A.J., Li H., Nelson H.M., Giorgio T.D., Duvall C.L. (2013). Ex vivo red blood cell hemolysis assay for the evaluation of pH-responsive endosomolytic agents for cytosolic delivery of biomacromolecular drugs. J. Vis. Exp..

[67] Ates G., Vanhaecke T., Rogiers V., Rodrigues R.M. (2017). Assaying Cellular Viability Using the Neutral Red Uptake Assay. Methods Mol. Biol..

[68] Carmichael J., DeGraff W.G., Gazdar A.F., Minna J.D., Mitchell J.B. (1987). Evaluation of a tetrazolium-based semiautomated colorimetric assay: Assessment of chemosensitivity testing. Cancer Res..

[69] Kumar P., Nagarajan A., Uchil P.D. (2018). Analysis of Cell Viability by the Lactate Dehydrogenase Assay. Cold Spring Harb. Protoc..

