# Supplementary material for: Heterobasidion annosum Induces Apoptosis in DLD-1 Cells and Decreases Colon Cancer Growth in In Vivo Model
Source: Int J Mol Sci. 2020 May 13;21(10):3447. doi: 10.3390/ijms21103447 (PMC7279362; doi:10.3390/ijms21103447)
Supplement: Supplementary file 1 [file ijms-21-03447-s001.pdf]

Title: Heterobasidion annosum induces apoptosis in DLD-1 cells and decreases colon cancer growth in in vivo model.

Anna Sadowska, Ewa Zapora, Diana Sawicka, Katarzyna Niemirowicz–Laskowska, Arkadiusz Surażyński, Katarzyna Sułkowska-Ziaja, Katarzyna Kała, Marcin Stocki, Marek Wołkowycki, Sławomir Bakier, Anna Pawlik, Magdalena Jaszek, Bożena Muszyńska, Halina Car

Supplementary Materials:

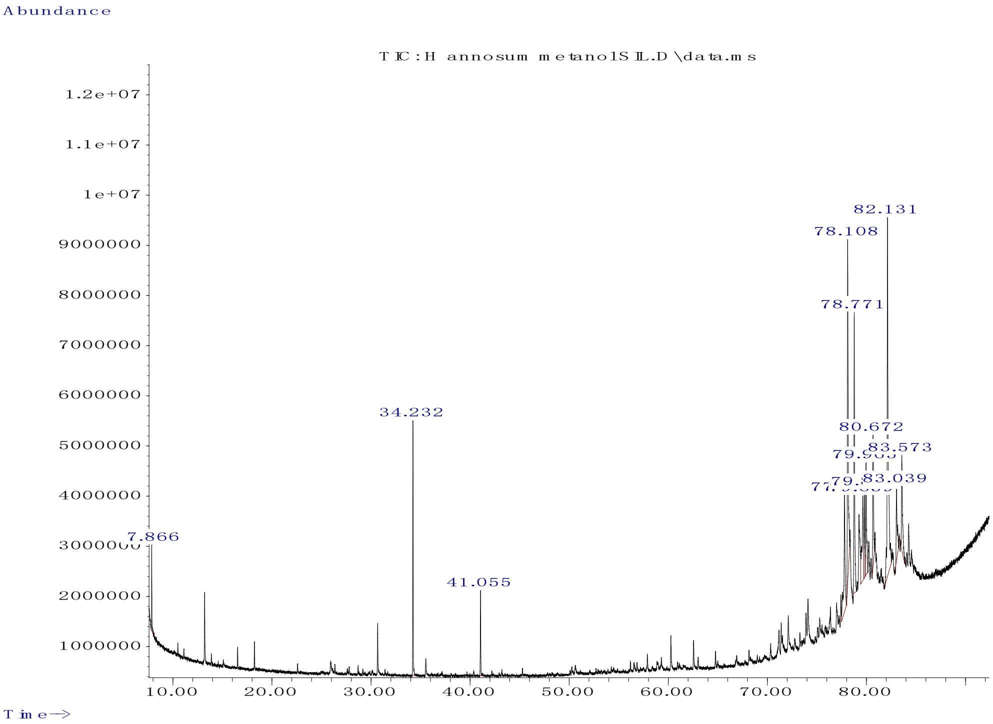

Figure S1. GC-MS chromatogram of chemical composition of *Heterobasidion annosum* fruiting bodies methanolic extract (% TIC, Total Ion Current).

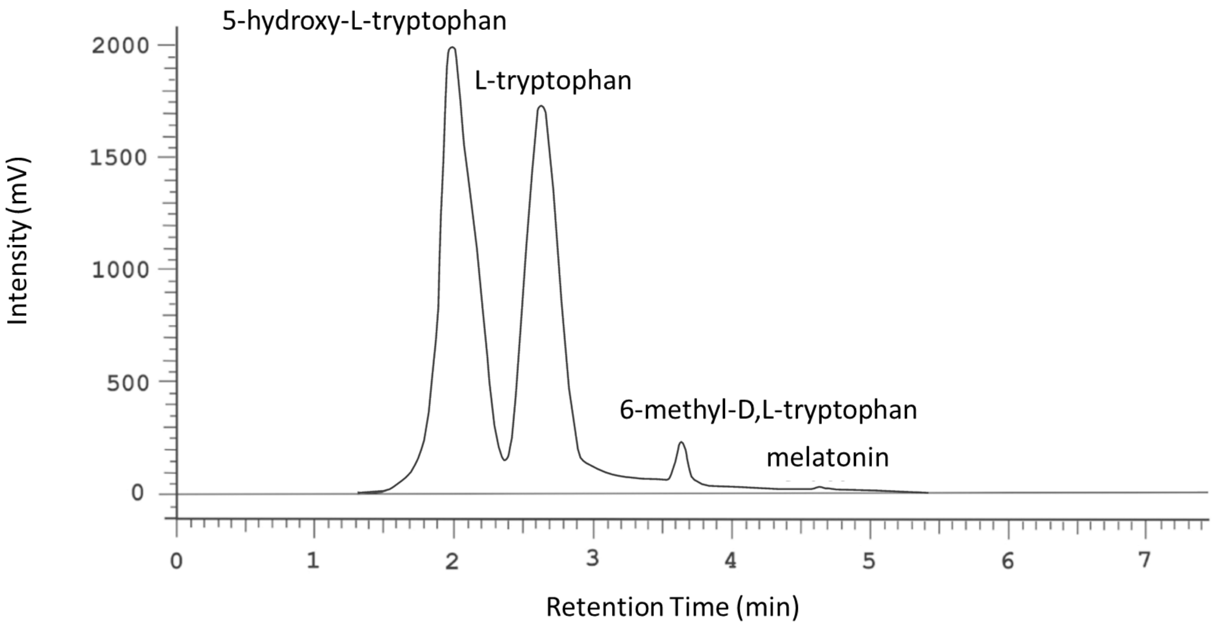

Figure S2. HPLC chromatogram of the analyzed indole compounds in *Heterobasidion annosum* extract.

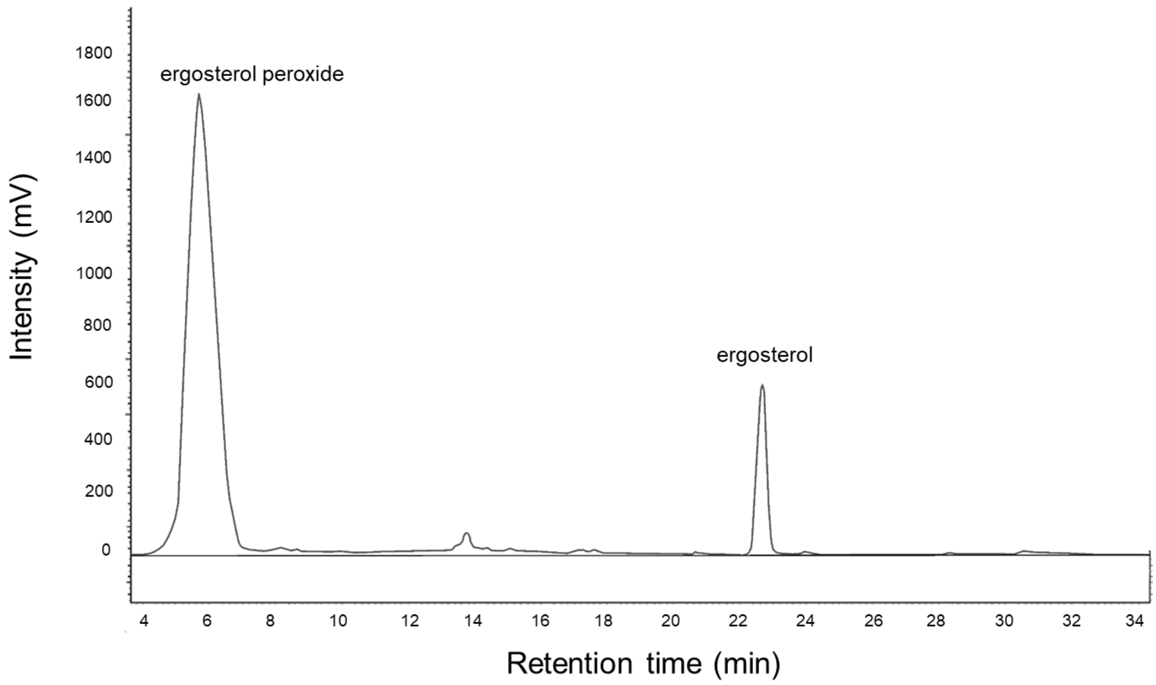

Figure S3. HPLC chromatogram of the analyzed sterols in *Heterobasidion annosum* extract.
